# Association of Peroxisome Proliferator-Activated Receptors (PPARs) with Diabetic Retinopathy in Human and Animal Models: Analysis of the Literature and Genome Browsers

**DOI:** 10.1155/2020/1783564

**Published:** 2020-03-03

**Authors:** Špela Tajnšek, Danijel Petrovič, Mojca Globočnik Petrovič, Tanja Kunej

**Affiliations:** ^1^University of Ljubljana, Biotechnical Faculty, Department of Animal Science, Slovenia; ^2^University of Ljubljana, Faculty of Medicine, Institute of Histology and Embryology, Slovenia; ^3^Eye Hospital, University Medical Center Ljubljana, Ljubljana, Slovenia; ^4^University of Ljubljana, Faculty of Medicine, Slovenia

## Abstract

Diabetic retinopathy (DR) is a condition that develops after long-lasting and poorly handled diabetes and is presently the main reason for blindness among elderly and youth. Peroxisome proliferator-activated receptors (PPARs) are nuclear receptors that are involved in carbohydrate and fatty-acid metabolism and have also been associated with DR. Three PPAR isoforms are known: *PPARG*, *PPARA*, and *PPARD*. In the present study, we retrieved articles reporting associations between PPARs and DR from PubMed database and compiled the data in two catalogues, for human and animal models. Extracted data was then complemented with additional relevant genomic information. Seven retrieved articles reported testing an association between *PPARs* with DR in human. Four of them concluded association of *PPARG* and *PPARA* with DR in European and Asian populations, having a protective role on DR development. One study reported pathogenic role of *PPARG*, while two articles reported no association between *PPARG* and DR among Indian and Chinese populations. Six retrieved articles reported testing of involvement of *PPARG* and *PPARA* in DR in animal models, including mouse and rat. The review includes case-control studies, meta-analysis, expression studies, animal models, and cell line studies. Despite a large number of documented sequence variants of the PPAR genes available in genome browsers, researchers usually focus on a small set of previously reported variants. Data extraction from Ensembl genome browser revealed several sequence variants with predicted deleterious effect on protein function which present candidates for further experimental validation. Results of the present analysis will enable more holistic approach for understanding of *PPARs* in DR development. Additionally, developed catalogues present a baseline for standardized reporting of PPAR-phenotype association in upcoming studies.

## 1. Introduction

Diabetic retinopathy (DR) is a condition that develops due to bad glycemic control in subjects with type 1 diabetes mellitus (T1DM) or type 2 diabetes mellitus (T2DM). Long-lasting poor blood glucose control, smoking, and hypertension can contribute to DR development [1, 2]. The disease progresses from nonproliferative (NPDR) to proliferative (PDR) stage where at first microvascular irregularities such as hemorrhage, ischemia, and microaneurysms lead to neoangiogenesis [2]. Microvascular changes start due to lower concentrations of oxygen in the retina of the eye after the disease progresses, and at final stages, PDR can lead to vision loss. Diabetic retinopathy had become the main reason for blindness in American adults. In year 2012, there were approximately 93 million people living with diabetic retinopathy, 17 million with PDR, and 21 with diabetic macular edema, and the number is expected to increase in the future [3, 4].

Peroxisome proliferator-activated receptors (PPARs) are nuclear receptors that regulate the expression of several genes and are affecting lipid and carbohydrate metabolism. PPARs consist of three subtypes: PPARA, PPARD, and PPARG [5]. Peroxisome proliferator-activated receptor gamma (*PPARG*) also known as *GLM1*, *CIMT1*, *NR1C3*, *PPARG1*, *PPARG2*, or *PPARgamma* is a nuclear receptor that binds hypolipidemic drugs and unsaturated fatty acids and affects adipocyte differentiation, gluconeogenesis, oxidation of fatty acids, lipogenesis, cholesterol metabolism, and synthesis of ketone bodies [5–9]. The gene is located on chromosome HSA3. In the eye, the gene is heterogeneously expressed in photoreceptor outer segments, choriocapillaries, retina, retinal pigmented epithelium, cornea, and lacrimal gland [10–12]. Three RNA isoforms of expressed *PPARG* have been identified: *γ*1, *γ*2, and *γ*3. PPAR-*γ*2 protein has additional stretch of 28 amino acids on N-terminal, and this extension seems to change PPAR-*γ*2 sensitivity to insulin action [13]. Proline variant of Pro12Ala (rs1801282) polymorphism of the *PPARG* gene is associated with increased resistance to insulin action whereas the alternative allele has the opposite properties [14].

Peroxisome proliferator-activated receptor alpha (*PPARA*) also known as *PPARα*, *NR1C1*, *hPPAR*, or *PPARalpha* is responsible for ketogenesis, lipid transport, lipogenesis, cholesterol metabolism, fatty acid transport, and oxidation [15]. It is located on the HSA22. *PPARA* is expressed in the retina; however, its levels have been shown to be reduced in the retinas with DR [16, 17]. Decreased *PPARA* expression in diabetic retinas contributes to retinal inflammation and neovascularization in DR, and activation of PPARA has anti-inflammatory and antiapoptotic effects in oxygen-induced retinopathy (OIR) and diabetic animal models through suppression of *NF*-*κB* signaling [16, 17].

Peroxisome proliferator-activated receptor delta (PPARD) also known as *FAAR*, *NUC1*, *NUCI*, *NR1C2*, *NUCII*, or *PPARB* is located on HSA6. It affects fatty acid transport and oxidation, adipocyte differentiation, adaptive thermogenesis, cell survival, and ubiquitination [18]. Among the three PPAR subtypes, it is the least studied and understood, especially its effects on inflammation and proliferation associating DR.

To our knowledge, the complete database related with reported associations between PPARs and DR does not yet exist. The aim of this study was therefore to conduct an overview of articles reporting an association between three PPARs and DR/PDR in human and animal models.

## 2. Materials and Methods

**Figure 1:**
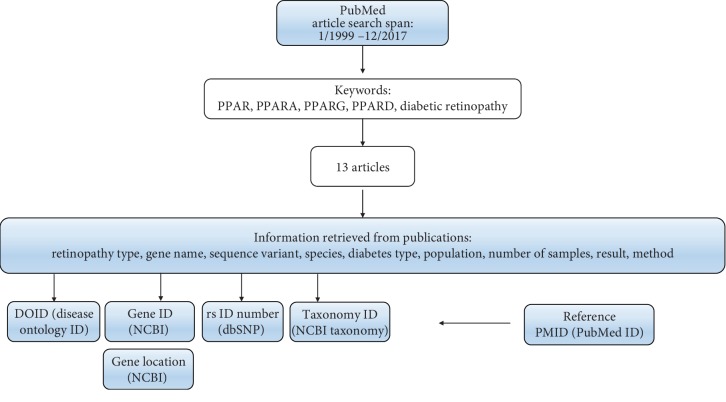
Workflow of the study.

Using keywords “*PPAR*” and/or “*PPARG*” and/or “*PPARA*” and/or “*PPARD*” and/or “polymorphism” and/or “diabetic retinopathy”, we explored the PubMed database for articles describing association between PPARs and DR in human and animal models. Inclusion criteria for the type of study in humans were case-control study, meta-analysis, or expression study. Retrieved articles included in previously published meta-analysis were excluded from the analysis. Time span for article search was set from January 1999 to December 2017. Retrieved articles were checked for the following information: retinopathy type, sequence variant, gene name, diabetes type, species, number of tested samples, result of the study, and method. The data extracted from publications was afterwards complemented with additional information such as gene ID (https://www.ncbi.nlm.nih.gov/gene), gene location (https://www.ncbi.nlm.nih.gov/gene), taxonomy ID (https://www.ncbi.nlm.nih.gov/taxonomy), disease ontology ID (DOID; http://disease-ontology.org/), reference SNP (rs) identification number, PubMed identification number (PMID) of the reference, and statistical significance (Figure 1). Ensembl genome browser release 96 was used to retrieve additional information related with sequence variants, predicted effect on protein function using six bioinformatics tools, and clinical significance from ClinVar database [19].

## 3. Results

**Figure 2:**
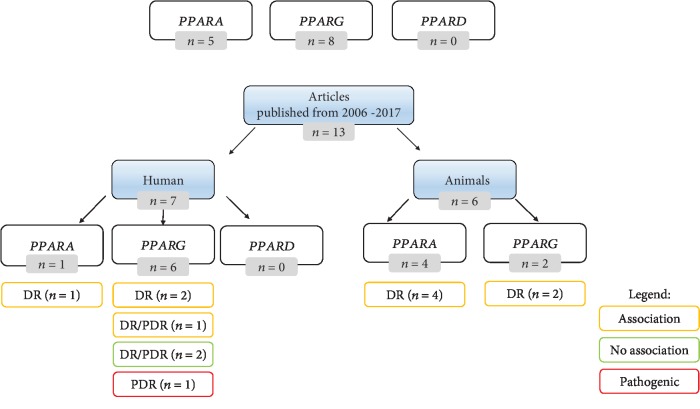
Overview of the results. The number of retrieved articles associating PPARs with DR/PDR is marked in grey color. Yellow color represents the number of articles reporting gene-DR/PDR association including protective effect or decreased DR risk. Green color represents the number of articles reporting no association between the gene and DR/PDR, and red color represents the number of articles reporting a pathogenic effect on DR/PDR.

We developed two tables consisting of data extracted from 13 retrieved articles published between 1/2012 and 12/2017 reporting associations between PPAR polymorphisms and DR in human (Table 1) and animal models (Table 2) (Figure 2). In humans, six articles reported testing association between *PPARG* and DR/PDR and one reported *PPARA* and DR association. We did not retrieve any articles related with *PPARD*-DR association. Six studies were performed in animal models, including four articles describing involvement of *PPARA* in DR and two involvement of *PPARG* in DR.

### 3.1. Studies in Humans

Out of seven retrieved articles describing association between *PPARs* and DR/PDR in humans, six articles were related with the *PPARG* gene and one study with the *PPARA* gene.

One study reported that *PPARG* may play an important role in the pathogenesis of PDR. The *PPARG* concentrations in the aqueous humor and vitreous fluid were significantly higher in PDR patients than in controls, and the level of *PPARG* increased in the advanced clinical stage. Additionally, a correlation between *PPARG* and vascular endothelial growth factor (*VEGF*) concentrations was identified [20]. Two out of seven studies reported no association between *PPARs* and DR [21, 22]. Three studies identified association (protective effect or decreased DR risk) between *PPARG* and DR/PDR (Figure 2) [23–25]. Qi et al. studied polymorphism rs1800206 of the *PPARA* gene and concluded that carriers of homozygous mutant allele have decreased DR risk in comparison to wild-type homozygotes in Chinese Han population [26].

Most participants in the studies had type 2 diabetes mellitus (T2DM), and some participants had type 1 diabetes mellitus (T1DM). The developed catalogue includes five case-control studies, one meta-analysis [23], and one expression study [20]. Case-control studies included 17 to 812 participants. Meta-analysis study consisted of more than 4000 participants from eight studies. Studies were performed on different populations, such as European Caucasian, Asian (Chinese Han), and Pakistani. Methods used for genotyping and expression analysis were quantitative real time, PCR-ligase detection reaction (LDR), quantitative PCR, PCR-RFLP, and real-time PCR.

### 3.2. Studies in Animal Models

Six studies used animal models for testing association between *PPARs* and DR/PDR: mouse, rat, and cattle. In some studies, more than one animal model and additional animal cell lines were used. For imitating DR or diabetes in mice and rat, animals were made diabetic with streptozotocin (STZ) or underwent through OIR. Most studies based on an animal model used knockout mice (KO) approach. In most studies, they used C57Bl/6J mouse model or Brown Norway rats [16, 17, 27–30]. Additionally, bovine retinal endothelial cells (BRECs) were also used [28].

Various methods were used for testing association between PPARs and DR in animal models, for example, TUNEL assay, quantitative real-time PCR, retinal leakage assay, vascular leakage assay, fluorescent microscopy, immunofluorescence, western blot, and protein-based detection methods detecting over/underexpression of the protein.

Most of the reports in humans were designed as association studies between *PPAR* polymorphisms and DR; however, in animal models, most performed gene expression analyses in diabetic and nondiabetic animals (Table 2). Hu et al. [17] used animal model for testing an involvement of *PPARA* and DR and concluded that *PPARA* knockout mice developed more severe DR which resulted in retinal vascular leakage, leukostasis, pericyte loss, capillary degeneration, and overexpressed inflammatory factors, whereas *PPARA* overexpression reduced vascular leakage and inflammation. *PPARA* protective effects have been proven by Ding et al. [30]. *PPARG*+/- knockout mice had greater leukostasis and leakage than wild-type mice [27], and suppression of *PPARG* has been shown to be involved in the pathogenesis of diabetic retinopathy and OIR [28].

## 4. Discussion

PPARs are important factors in DR/PDR due to their protective function on the disease development. Our results revealed that reports in this study field are very heterogeneous. Most studies in humans analyzed polymorphism Pro12Ala (rs1801282) located in the *PPARG* gene. In contrary, some studies were performed on cell lines and animal models. For example, Chen et al. [29] reported that *PPARA* is a target of microRNA-21, which downregulates expression of *PPARA* and worsens DR condition.

Our study revealed that researchers use different synonyms for the same gene (for example, *PPARG*, *PPARγ*, *CIMT1*, and *NR1C3*), for the same gene variant (Pro12Ala, rs1801282, c.34C>G), or for methodology. In several studies, patients with DR were not divided into NPDR and PDR cases. Additionally, in some studies, it is not clear whether a gene is associated with PDR or is associated only with NPDR.

The results of the association studies related with PPARs and its association with DR/PDR differ among populations (Table 1). For example, polymorphism Pro12Ala is the most studied polymorphism of the *PPARG* gene. Tariq et al. reported that polymorphism Pro12Ala is not associated with DR in Pakistani population [25]; however, Wang et al. reported that it is associated with DR in Chinese population [24].

**Figure 3:**
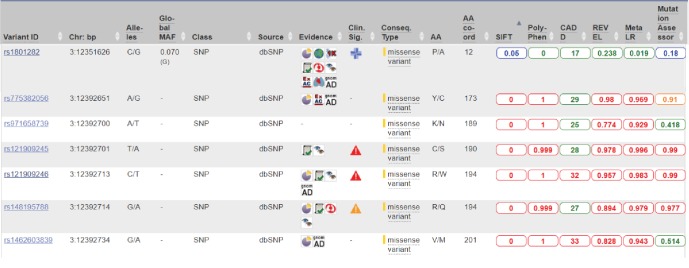
Print screen of the Ensembl genome browser presenting a part of the variant table of the PPARG gene. The table includes seven selected sequence variants and results of bioinformatics prediction of their effect on protein function. Ensembl browser includes six tools for predicting effects of substitutions on protein function: SIFT, PolyPhen, CADD. REVEL, MetaLR, and Mutation Assessor. Predicted effect on protein function is shown in different colors: benign: green; tolerated/neutral: blue; possibly damaging: orange; damaging: red. Clin. Sig.: clinical significance; a classification of a variant's impact on disease, according to the ClinVar database: pathogenic: red triangle; likely pathogenic: orange triangle; likely benign: blue cross.

According to the latest release of the Ensembl database, there are a high number of polymorphisms located within *PPAR* genes in humans and animals. However, our results show that researchers focused on only few sequence variants of the *PPAR* gene family. Several bioinformatics tools could be used for prioritization of stronger candidate sequence variants for experimental validation. Ensembl browser enables comparison of six bioinformatics tools designed for predicting the effect of sequence variants on protein function: SIFT, PolyPhen, CADD. REVEL, MetaLR, and MutationAssessor.  Figure 3 presents a part of the variant table from the Ensembl genome browser. For example, most of the tools predict benign effect of the polymorphism rs1801282 (Pro12Ala) on protein function and two predict tolerated/neutral effect. On the contrary, for several other polymorphisms, predicted effect on protein function is damaging (red color) or possibly damaging (orange). Out of 286 sequences with available bioinformatics predictions, only polymorphism rs121909246 has predicted deleterious effect by all six bioinformatics tools. Additionally, according to the ClinVar database, this polymorphism has a pathogenic effect. However, several other missense polymorphisms of the *PPARG* gene have not yet been tested for association with diseases, including DR. For some of the polymorphisms, minor allele frequency (MAF) and clinical significance from ClinVar database are given. Currently, the Ensembl browser lists 10 sequence variants of the *PPARG* gene with pathogenic clinical effect extracted from the ClinVar database. Further bioinformatics prioritization studies should be performed for all three *PPAR* genes to select novel candidate loci for functional analyses. However, it should be noted that bioinformatics predictions obtained using these tools often differ and are not always consistent with results of experimental validation.

Several other approaches were also used for testing involvement of PPAR in DR and development of novel therapies [16, 31]. Dou et al. analyzed substances targeting PPARs in association with DR/PDR [32]. Human cell lines were used as model for testing the association between gene and disease [17, 30, 33]. Many drugs such as fibrates and thiazolidinediones have been widely used for treatment of dyslipidemia and have also been found to have direct association with PPARs. Fibrates are amphipathic carboxylic acids, and its therapeutic effects are *PPARA* dependent, which makes fibrates selective agonists of *PPARA*. Chen et al. [34] provided evidence of association between fibrates and *PPARA* with different *PPARA* agonists, *PPARA* antagonists, and *PPARA-/-* knockout mice and stated that fenofibrates work as *PPARA* activators which leads to transcription activation or inhibition of *PPARA* target genes which further leads to slower DR progression. Rosiglitazone, an antidiabetic drug from thiazolidinedione class, works as a *PPARG* agonist, and its work of action has been studied in diabetic mice [27].

Besides members of the *PPAR* family, several other genes have been tested in association with DR development, for example, *PPARGC1* and *VEGF* [35, 36]. Therefore, catalogues developed in the present study should be extended with additional genes, tested for association with DR. For future publications, it would be suggested to present the data with an official gene name and with rs ID name of the polymorphism. It is also suggested that researchers clearly state if the gene is associated with DR, PDR, or NPDR. Standardized reporting of genotype-phenotype is important for further research on this prominent topic for easier overview on the data to make new associations on PPARs and DR development and finding new targets for DR treatment.

## 5. Conclusions

PPARs are important protective factors of DR/PDR among certain populations and have potential for therapeutic targets. To the best of our knowledge, this is the first overview on the topic on PPARs associated with DR/PDR in human and animal models. The study presents a baseline for further studies, for example, meta-analyses and bioinformatics prioritization of new candidates for functional studies.

## Figures and Tables

**Table 1:** Summary of extracted data from studies reporting association between *PPAR* genes and DR/PDR in humans.

Gene symbol	Gene ID	Gene location	Sequence variant	rs ID of the polymorphism	Diabetes type	Retinopathy type	DOID	Population	Number of samples (cases/controls)	Statistical significance	Method	Main result of the study	Type of study	Reference	PMID
*PPARG*	5468	3p25.2	/	/	T1DM T2DM	PDR	13207	Japan^∗^	17 (12 PDR, 5 controls)	*p* < 0.0005	Quantitative real-time PCR, ELISA, immunohistochemistry analysis	Higher expression of *PPARG* in PDR versus controls	Expression study	Katome et al. [20]	25468312
*PPARG*	5468	3p25.2	rs1801282 rs3856806 rs12497191	rs1801282 rs3856806 rs12497191	T2DM	DR, PDR	8947, 13207	Chinese	792 T2DM (448 DR, 344 diabetes without DR)	OR (95% CI) dominant model GG: 1.40 (0.85-2.29); *p* = 0.22	PCR-LDR	No significant association between polymorphisms in the *PPARG* gene and DR or PDR	Case-control study	Zhang et al. [22]	25274455
*PPARG*	5468	3p25.2	Pro12Ala	/	T2DM	DR	8947	Caucasian Asian	5170 (2720 DR cases, 2450 controls)	Caucasian subgroup (OR = 0.74; 95% CI: 0.59-0.94, *p* = 0.01) Asian subgroup (OR = 0.77; 95% CI: 0.55-1.07, *p* = 0.12)	Statistics	Protective effect of Pro12Ala on DR in T2DM with ethnic differences	Meta-analysis	Ma et al. [23]	22993484
*PPARG*	5468	3p25.2	C1341T Intron A>C Pro12Ala Intron C>T	rs3856806 rs709158 rs1805192 rs4684847	T2DM	DR	8947	Chinese	500 T2DM (247 DR cases, 253 controls)	OR (95%CI) = 0.86 (0.65-0.96), *p* = 0.012	Quantitative PCR	rs1805192 minor allele (Ala) of *PPARG* is significantly associated with lower DR risk; combined effect of Ala-BMI interaction between polymorphism and overweight on DR	Case-control study	Wang et al. [24]	26885119
*PPARG*	5468	3p25.2	rs1801282 (c.34C>G, Pro12Ala)	rs1801282	T2DM	DR, PDR	8947, 13207	Pakistani	573 (189 DR, 193 DNR, 200 controls)	OR = 0.4; 95%CI = 0.2‐0.8	PCR-RFLP	Protective role of the 12Ala polymorphism against PDR in T2DM	Case-control study	Tariq et al. [25]	23559865
*PPARG*	5468	3p25.2	p.Pro12Ala	/	T2DM	DR	8947	Indian	1325 (717 DR, 608 T2DM without DR)	*p* = 0.507	Real-time PCR	No significant association	Case-control study	Kaur et al. [21]	27427939
*PPARA*	5465	22q13.31	rs4253778 rs135539 rs1800206	rs4253778 rs135539 rs1800206	T2DM	DR	8947	Chinese Han	812 (402 DR, 410 control)	OR (95%CI) = 0.78 (0.66-0.94)	Quantitative PCR	Association between rs1800206 minor (V) allele and lower risk for DR; interaction between rs1800206 and abdominal obesity	Case-control study	Qi et al. [26]	26671228

/ = data not available; ^∗^ the country where the study was conducted; PPARA = peroxisome proliferator-activated receptor alpha; PPARG = peroxisome proliferator-activated receptor gamma; PPARD = peroxisome proliferator-activated receptor delta; DR = diabetic retinopathy; PDR = proliferative diabetic retinopathy; DOID = disease ontology ID; PMID = PubMed ID; T1DM = type 1 diabetes mellitus; T2DM = type 2 diabetes mellitus; DNR = diabetes no retinopathy; LDR: ligase detection reaction.

**Table 2:** Summary of extracted data from studies reporting involvement of *PPAR* genes in diabetic retinopathy in animal models.

Gene symbol	Gene ID	Gene location	Species	Taxonomy ID	Sequence variant	Model	Retinopathy type model	DOID	Strain/details	Statistical significance	Method	Main result of the study	Type of study	Reference	PMID
*Pparg*	19016	6 E3	Mouse	10090	/	Knockout, STZ	DR	8947	C57BL/6 PPARg(+/-)	*p* < 0.05	Retinal leakage assay, fluorescent microscopy	*Pparg* signaling pathway inhibits diabetes-inducedretinal leukostasis and leakage	Animal model	Muranaka et al. [27]	17003451
*Pparg*	25664	4q42	Rat	10116	/	STZ	DR	8947	Brown Norway	*p* < 0.05	Retinal leakage assay, fluorescent microscopy	Therapy with *Pparg* ligands may inhibit retinal leukostasis and retinal leakage in diabetes	Animal model	Muranaka et al. [27]	17003451
*Pparg*	19016	6 E3	Mouse	10090	/	Knockout, STZ, OIR	DR	8947	C57BI/6J	*p* < 0.05	Immunofluorescence, western blot	The link between *Pparg* and retinal vascular inflammation in DR	Animal model	Tawfik et al. [28]	18806296
*PPARG*	281993	22q24	Cattle	9913	/	Cells	DR	8947	BRECs	*p* < 0.05	Western blot	Suppression of *Pparg* expression in high glucose-treated cells	Animal model	Tawfik et al. [28]	18806296
*Ppara*	19013	15 E2	Mouse	10090	/	Knockout, OIR, cells	DR	8947	C57BLKS/J C57BL/6J	*p* < 0.05	qRT-PCR	Upregulated *miR-21* and downregulated *Ppara* in OIR	Cell line, animal model	Chen et al. [29]	28270521
*Ppara*	19013	15 E2	Mouse	10090	/	Knockout, OIR	DR	8947	Ppara(-/-) C57/BL6J	*p* ≤ 0.05	TUNEL assay	Y-0452 exerts antiangiogenic effects in OIR retinas through *Ppara*-dependent mechanism	Cell line, animal model	Deng et al. [16]	28979999
*Ppara*	25747	7q34	Rat	10116	/	STZ	DR	8947	Brown Norway	*p* ≤ 0.05	Vascular leakage assay	Y-0452 (*Ppara* agonist) alleviated the retinal apoptosis	Cell line, animal model	Deng et al. [16]	28979999
*Ppara*	19013	15 E2	Mouse	10090	/	Knockout, STZ	DR	8947	Ppara(-/-) C57/BL6J	*p* < 0.05	TUNEL assay	Protective effect of *Ppara* against retinal pericyte loss in DR	Animal model	Ding et al. [30]	25108226
*Ppara*	19013	15 E2	Mouse	10090	/	Knockout, STZ	DR	8947	C57BL/6J PPARA knockout Akita db/db	*p* < 0.05	Quantitative real-timeRT-PCR	*Ppara* knockout mice developed more severe DR	Animal model	Hu et al. [17]	24003152
*Ppara*	25747	7q34	Rat	10116	/	STZ	DR	8947	Brown Norway	*p* < 0.05	Quantitative real-timeRT-PCR	Overexpression of *Ppara* in the retina alleviated vascular leakage and inflammation	Animal model	Hu et al. [17]	24003152

/ = data not available; Pparg = peroxisome proliferator-activated receptor gamma; Ppara = peroxisome proliferator-activated receptor alpha; STZ = streptozotocin; OIR = oxygen-induced retinopathy; DR = diabetic retinopathy; BREC = bovine retinal endothelial cells; DOID = disease ontology identification number.

## Additional Points

*Executive Summary*. (i) Literature review of studies testing an association between PPARs and diabetic retinopathy in human and animal models. (ii) Results showed that published results are opposing and data presentation of results in publications is heterogeneous. (iii) Developed catalogues summarizing PPAR-DR associations present a baseline for standardized reporting of PPAR-phenotype association in upcoming studies. (iv) Prioritization of novel candidate sequence variants for further experimental validation using six bioinformatics tools revealed several substitutions with predicted deleterious effect on protein function.

## References

[1] Molla G. J., Ismail-Beigi F., Larijani B. (2019). Smoking and diabetes control in adults with type 1 and type 2 diabetes: a nationwide study from the 2018 National Program for Prevention and Control of Diabetes of Iran. *Canadian Journal of Diabetes*.

[2] Liew G., Klein R., Wong T. Y. (2009). The role of genetics in susceptibility to diabetic retinopathy. *International Ophthalmology Clinics*.

[3] Yau J. W. Y., Rogers S. L., Kawasaki R. (2012). Global prevalence and major risk factors of diabetic retinopathy. *Diabetes Care*.

[4] Lee R., Wong T. Y., Sabanayagam C. (2015). Epidemiology of diabetic retinopathy, diabetic macular edema and related vision loss. *Eye and Vision*.

[5] The UniProt Consortium (2019). UniProt: a worldwide hub of protein knowledge. *Nucleic Acids Research*.

[6] Issemann I., Green S. (1990). Activation of a member of the steroid hormone receptor superfamily by peroxisome proliferators. *Nature*.

[7] Finck B. N. (2007). The PPAR regulatory system in cardiac physiology and disease. *Cardiovascular Research*.

[8] Duan S. Z., Usher M. G., Mortensen R. M. (2009). PPARs: the vasculature, inflammation and hypertension. *Current Opinion in Nephrology and Hypertension*.

[9] Wang Y.-X. (2010). PPARs: diverse regulators in energy metabolism and metabolic diseases. *Cell Research*.

[10] Herzlich A. A., Tuo J., Chan C.-C. (2008). Peroxisome proliferator-activated receptor and age-related macular degeneration. *PPAR Research*.

[11] Beauregard C., Brandt P. C. (2003). Peroxisome proliferator-activated receptor agonists inhibit interleukin-1beta-mediated nitric oxide production in cultured lacrimal gland acinar cells. *Journal of Ocular Pharmacology and Therapeutics*.

[12] Sarayba M. A., Li L., Tungsiripat T. (2005). Inhibition of corneal neovascularization by a peroxisome proliferator- activated receptor-*γ* ligand. *Experimental Eye Research*.

[13] Yen C.-J., Beamer B. A., Negri C. (1997). Molecular scanning of the human peroxisome proliferator activated receptor *γ* (hPPAR*γ*) gene in diabetic Caucasians: identification of a Pro12Ala PPAR*γ*2 missense mutation. *Biochemical and Biophysical Research Communications*.

[14] Hara K., Okada T., Tobe K. (2000). The Pro12Ala Polymorphism in PPAR*γ*2 May Confer Resistance to Type 2 Diabetes. *Biochemical and Biophysical Research Communications*.

[15] Leone T. C., Weinheimer C. J., Kelly D. P. (1999). A critical role for the peroxisome proliferator-activated receptor *α* (PPAR*α*) in the cellular fasting response: the PPAR*α*-null mouse as a model of fatty acid oxidation disorders. *Proceedings of the National Academy of Sciences of the United States of America*.

[16] Deng G., Moran E. P., Cheng R. (2017). Therapeutic effects of a novel agonist of peroxisome proliferator-activated receptor alpha for the treatment of diabetic retinopathy. *Investigative Opthalmology & Visual Science*.

[17] Hu Y., Chen Y., Ding L. (2013). Pathogenic role of diabetes-induced PPAR-*α* down-regulation in microvascular dysfunction. *Proceedings of the National Academy of Sciences of the United States of America*.

[18] Kanehisa M., Furumichi M., Tanabe M., Sato Y., Morishima K. (2017). KEGG: new perspectives on genomes, pathways, diseases and drugs. *Nucleic Acids Research*.

[19] Zerbino D. R., Achuthan P., Akanni W. (2018). Ensembl 2018. *Nucleic Acids Research*.

[20] Katome T., Namekata K., Mitamura Y. (2015). Expression of intraocular peroxisome proliferator-activated receptor gamma in patients with proliferative diabetic retinopathy. *Journal of Diabetes and its Complications*.

[21] Kaur N., Vanita V. (2016). Association analysis of PPAR*γ* (p.Pro12Ala) polymorphism with type 2 diabetic retinopathy in patients from North India. *Ophthalmic Genetics*.

[22] Zhang Y., Meng N., Lv Z., Li H., Qu Y. (2015). The gene polymorphisms of UCP1 but not PPAR *γ* and TCF7L2 are associated with diabetic retinopathy in Chinese type 2 diabetes mellitus cases. *Acta Ophthalmologica*.

[23] Ma J., Li Y., Zhou F., Xu X., Guo G., Qu Y. (2012). Meta-analysis of association between the Pro12Ala polymorphism of the peroxisome proliferator–activated receptor-*γ*2 gene and diabetic retinopathy in Caucasians and Asians. *Molecular Vision*.

[24] Wang Y., Wang X. H., Li R. X. (2015). Interaction between peroxisome proliferator-activated receptor gamma polymorphism and overweight on diabetic retinopathy in a Chinese case-control study. *International Journal of Clinical and Experimental Medicine*.

[25] Tariq K., Malik S. B., Ali S. H. B. (2013). Association of Pro12Ala polymorphism in peroxisome proliferator activated receptor gamma with proliferative diabetic retinopathy. *Molecular Vision*.

[26] Qi S., Wang C., Zhang Y., Cheng Y., Wang S., Zhao Y. (2016). The association of peroxisome proliferator-activated receptor *α* with diabetic retinopathy, and additional gene-obesity interaction in Chinese type 2 diabetes mellitus patients. *Obesity Research & Clinical Practice*.

[27] Muranaka K., Yanagi Y., Tamaki Y. (2006). Effects of peroxisome proliferator-activated receptor *γ* and its ligand on blood–retinal barrier in a streptozotocin-induced diabetic model. *Investigative Opthalmology & Visual Science*.

[28] Tawfik A., Sanders T., Kahook K., Akeel S., Elmarakby A., Al-Shabrawey M. (2009). Suppression of retinal peroxisome proliferator-activated receptor *γ* in experimental diabetes and oxygen-induced retinopathy: role of NADPH oxidase. *Investigative Opthalmology & Visual Science*.

[29] Chen Q., Qiu F., Zhou K. (2017). Pathogenic role of microRNA-21 in diabetic retinopathy through downregulation of PPAR*α*. *Diabetes*.

[30] Ding L., Cheng R., Hu Y. (2014). Peroxisome proliferator-activated receptor *α* protects capillary pericytes in the retina. *The American Journal of Pathology*.

[31] Behl T., Kaur I., Goel H., Kotwani A. (2016). Implications of the endogenous PPAR-gamma ligand, 15-deoxy-delta-12, 14-prostaglandin J2, in diabetic retinopathy. *Life Sciences*.

[32] Dou X. Z., Nath D., Shin Y., Ma J. X., Duerfeldt A. S. (2018). Structure-guided evolution of a 2-phenyl-4-carboxyquinoline chemotype into PPAR*α* selective agonists: new leads for oculovascular conditions. *Bioorganic & Medicinal Chemistry Letters*.

[33] Savage S. R., Bretz C. A., Penn J. S. (2015). RNA-Seq reveals a role for NFAT-signaling in human retinal microvascular endothelial cells treated with TNF*α*. *PLoS One*.

[34] Chen Y., Hu Y., Lin M. (2013). Therapeutic effects of PPAR*α* agonists on diabetic retinopathy in type 1 diabetes models. *Diabetes*.

[35] Petrovic M. G., Kunej T., Peterlin B., Dovc P., Petrovic D. (2005). Gly482Ser polymorphism of the peroxisome proliferator-activated receptor-gamma coactivator-1 gene might be a risk factor for diabetic retinopathy in Slovene population (Caucasians) with type 2 diabetes and the Pro12Ala polymorphism of the PPARgamma gene is not. *Diabetes/Metabolism Research and Reviews*.

[36] Sajovic J., Cilenšek I., Mankoč S. (2019). Vascular endothelial growth factor (VEGF)-related polymorphisms rs10738760 and rs6921438 are not risk factors for proliferative diabetic retinopathy (PDR) in patients with type 2 diabetes mellitus (T2DM). *Bosnian Journal of Basic Medical Sciences*.

